# Analysis of Non-Stationarity for 5.9 GHz Channel in Multiple Vehicle-to-Vehicle Scenarios

**DOI:** 10.3390/s21113626

**Published:** 2021-05-23

**Authors:** Fang Li, Wei Chen, Yishui Shui

**Affiliations:** 1 School of Automation, Wuhan University of Technology, Wuhan 430070, China; lifang_zdh@whut.edu.cn (F.L.); greatchen@whut.edu.cn (W.C.); 2 Guangdong Communications and Networks Institute, Guangzhou 510700, China; 3 The 7th Research Institute of China Electronics Technology Group Corporation, Guangzhou 510310, China; 4 School of Communication and Information Engineering, South China University of Technology, Guangzhou 510641, China

**Keywords:** V2V, stationary time, channel measurement, wireless communication

## Abstract

The vehicle-to-vehicle (V2V) radio channel is non-stationary due to the rapid movement of vehicles. However, the stationarity of the V2V channels is an important indicator of the V2V channel characteristics. Therefore, we analyzed the non-stationarity of V2V radio channels using the local region of stationarity (LRS). We selected seven scenarios, including three directions of travel, i.e., in the same, vertical, and opposite directions, and different speeds and environments in a similar driving direction. The power delay profile (PDP) and LRS were estimated from the measured channel impulse responses. The results show that the most important influences on the stationary times are the direction and the speed of the vehicles. The average stationary times for driving in the same direction range from 0.3207 to 1.9419 s, the average stationary times for driving in the vertical direction are 0.0359–0.1348 s, and those for driving in the opposite direction are 0.0041–0.0103 s. These results are meaningful for the analysis of the statistical characteristics of the V2V channel, such as the delay spread and Doppler spread. Small-scale fading based on the stationary times affects the quality of signals transmitted in the V2V channel, including the information transmission rate and the information error code rate.

## 1. Introduction

In the past few years, 5G communication technology has tremendously progressed. Benefiting from the development of 5G communication technology, vehicle-to-vehicle (V2V) communication systems have attracted considerable research interest, with many achievements in this area [1,2]. The industry has implemented many standards for V2V communication, for instance, the IEEE 802.11 p protocol introduced by IEEE [3] and the LTE-V promoted by the Ministry of Industry [4]. Thus, it can be seen that V2V communication is an important aspect in 5G and 6G communication around the world. The development trend in 5G and 6G communication is that the channel environment of communication equipment is changing from stationary channels to non-stationary channels. Traditional channel models, such as the cost 207 [5] and channel models in channel simulators, are stationary channel models that include a fixed multipath number, a fixed multipath delay, and a Doppler power spectrum for each delay. This stationary channel model is not applicable in 5G and 6G.

To evaluate the non-stationary wireless channel, channel modeling and channel measurement [6] are usually used. Channel modeling is divided into geometry-based stochastic channel modeling methods [7,8] and deterministic channel modeling based on the ray tracing method [9,10]. However, the propagation environment of electromagnetic waves is ever-changing. To describe channel characteristics more exactly, channel measurement is a required task, so various international organizations have undertaken considerable work in this area; the measured propagation channel models for 5G-NR/ IMT-2020 systems have resulted in the 3GPP standards [11,12].

Similarly, the measurement and modeling of vehicular wireless channels provide an important theoretical basis for research on vehicular communication technology. Many scholars have shown that the parameters of V2V channels, such as the root-mean-square delay spread [13], power delay profile (PDP) [14], and Doppler power spectrum [15], vary with time. Moreover, due to the random high-speed movement of the receiving and transmitting antennas in wireless vehicle communication and the existence of many high-speed moving scatters in the communication environment [16], the vehicle wireless channel presents obvious non-stationary characteristics. Therefore, it is necessary to know how long the channel can be considered to be constant. The maximum time interval for a time-varying channel to ensure wide stability is the channel stationary time, which describes the channel non-stationarity characteristics of the channel.

The wide-sense stationary uncorrelated scattering (WSSUS) assumption [17] was proposed to simplify the statistical characteristics of the channel. It has been widely used in the analysis of various cellular scenarios, such as device-to-device (D2D) communications and mobile crowdsensing [18,19]. As vehicular communication is one aspect of D2D communications, the statistical characteristics of V2V channels are meaningful to WSSUS. With the increase in research on the Internet of vehicles, the authors of [20] provided the definitions of stationary time and stationary bandwidth, which use the local scattering function (LSF) [21,22] and the channel correlation function (CCF). The LSF extends the scattering function [23] of WSSUS channels.

To evaluate the performance of the LSF method, mean square error (MSE) was used [21]. The results showed that there was an optimal combination of parameters in the local scattering function, and the minimum MSE decreased with the increase in the LSF until it reached a minimum and then increased again. Furthermore, the authors of [24] extended the research on the non-stationary characteristics of vehicular wireless channels from the time domain to the frequency domain. The results showed that the fading process of vehicular wireless channels had strong non-stationarity characteristics and correlation scattering. The minimum steady-state interval was about 40 ms in time and 40 MHz in frequency.

Many experimental studies have described the non-stationarity of the V2V channel over time. Some papers have already investigated the non-stationarity of V2V channels. For example, based on the vehicle wireless channel measurement in an expressway scenario, it was observed that the line of sight (LoS) propagation path changed rapidly in the delay Doppler domain [25]. Through the collinearity estimation of a time-dependent local scattering function sequence, the steady-state time was obtained. The results showed that the average steady-state time was about 23 ms in the expressway scenario, and 1479 ms in the same environment when two vehicles were driving in the same direction. For an urban scenario, the average steady-state time of two vehicles in the same direction was 1412 ms. Similarly, the authors of [26,27] evaluated the collinearity of this LSF sequence, which allowed for the quantification of the time interval over which the vehicular channel can be approximated as a WSSUS [28].

Therefore, the literature shows that the steady-state time of communication vehicles traveling in the same or in the opposite direction will differ greatly. In [25], spectral divergence was used to analyze the non-stationarity of vehicular channels; in [29], the complexity of spectral divergence was investigated based on V2V measurements. References [30,31] defined a statistical test based on the evolutionary spectrum of a signal estimated at different time instances. The US assumption has been tested far less [32]. A more traditional measure of the changes in channel statistics is the shadow-fading correlation [33,34]. Similarly, the time–frequency properties played a dominant role in the determination of quasi-stationarity regions in the distance [35]. Reference [35] mainly considered an urban macrocell scenario. In [36,37], the correlation matrix distance (CMD) was employed to characterize the non-stationarity of V2V MIMO channels. The time-varying WSS time window was estimated based on CMD in a suburban scene under a V2V environment with the threshold of $c_{th}=0.1,\;0.2$, and 0.3. The measurement campaigns in the above studies mainly considered the LoS condition. For non-line-of-sight (NLoS), References [38,39] studied both LoS and NLoS channels with the test cars driving in the same direction. They found that the stationarity distance in LoS cases was larger than that in NLoS cases because the main path energy in the LoS case was stronger, whereas the main path energy in the NLoS case was much smaller compared with the other scattering paths, resulting in a smaller stationarity distance of channel. However, the article only discussed the same direction condition.

The complex traffic environment in China is different from that in Europe and North America. There are four first-tier cities in China; the resident population of each is close to 20 million. Additionally, there are 15 new first-tier cities in China, and the population of each city ranges from 7 to 10 million. Hence, any of these cities would be a super city in developed countries. The high population density and the soaring number of vehicles characterize China’s traffic situation. Therefore, this city expansion has led to the complexity and diversity of China’s road network, and congestion has become a common phenomenon. The average speed of vehicles in 400 cities in China is generally around is 6.89 m/s, which is different from that in [39] (13.89 m/s). Therefore, to reduce congestion, many viaducts and expressways have been built in these cities. Meanwhile, the rapid urban expansion has led to rapid changes in road conditions. Some areas are developed with limited complex infrastructure, but others are densely populated residential areas or office areas. In particular, the southern Chinese cities are densely covered by lakes and rivers, so a large number of large-span bridges have become a major feature there. Here, some areas are open, and in others, vehicles enter residential areas or office areas with dense high-rise buildings.

To fill the gap in the literature, our method generalizes the approach proposed in [28], in which the local region of stationarity (LRS) was employed to characterize the non-stationarity of V2V channels.

In particular, the main contributions of this paper include:

- We obtained a large amount of high-precision data measured under various road conditions in China. We selected seven scenarios for comparison with each other to explore the effect of surroundings from the measured data. The statistical channel characteristics acquired by continuous measurements are more accurate.
- To determine the influence of relative speed, we select three typical driving directions of two vehicles: the same direction, the perpendicular direction, and the opposite direction, for comparison.
- We compare the characteristics of the PDP in different driving conditions. The differences in the scattering environment in different scenarios can be observed.
- The most important factors affecting the stationary time are the relative speed and the environment. The temporal PDP correlation coefficient is used to explain the non-stationarity phenomenon.

The rest of this paper is organized as follows: The parameters of the channel sounder and measurement setups are presented in Section 2. Section 3 illustrates the calculation of the local region of stationarity. The channel characteristics are interpreted in Section 4, whereas Section 5 draws the conclusions. In the end, a discussion of the results and future research needs is outlined in Section 6.

## 2. Measurements

### 2.1. Measurement Setup

**Figure 1:**
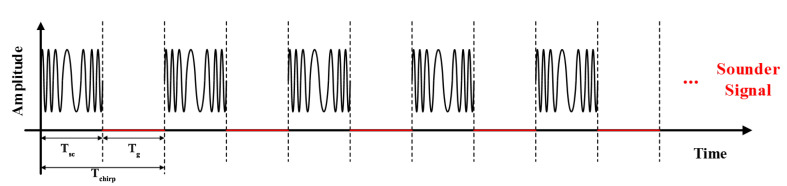
Structure of the transmitted signal.

**Figure 2:**
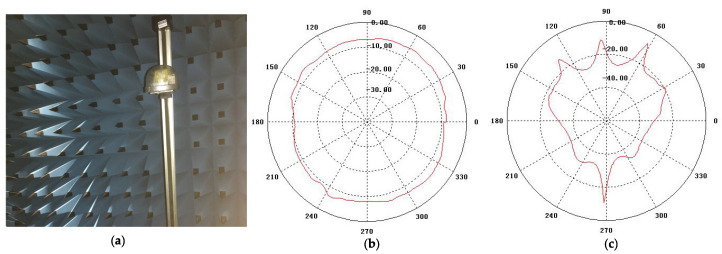
The antenna used in the measurement campaign. (**a**) Photo of the antenna, (**b**) the azimuth antenna pattern, and (**c**) the elevation antenna pattern.

The time-division multiplexing channel sounder used in measurement campaigns was provided by the Norwegian University of Science and Technology (NTNU) and Selskapet for Industriellog Teknisk Forskning (SINTEF) [40]. Compared with other channel sounders, it has the advantages of being small, easy portability, and a stable signal that can be carried in vehicles. The sampling frequency of the channel sounder can reach 1 GHz/s, and the sounder works at a center frequency of 5.9 GHz with a bandwidth of 100 MHz. Thus, the channel sounder delay resolution $\Delta \tau_{min}=10$ ns can be derived. The power $P_{Tx}$ of the transmitter (Tx) part is 16 dBm. We configured the Tx to transmit 1933 chirps in one second. The chirp signal with a period of $T_{chirp}$ = 517.24 s contains an interval of $T_{g}$ = 491.64 s and a signal length of $T_{sc}$ = 25.6 s. The structure of the transmitted signal can be found in Figure 1. The signal is emitted by an omnidirectional antenna (Figure 2). Before we conducted the measurement campaign, antennas with gains of 2 dBi were mounted on the apex of the vehicle roofs. The antenna pattern at 5.9 GHz is depicted in Figure 2b,c, in which we can observe that the antenna is omnidirectional in the horizontal plane (H-plane).

**Figure 3:**
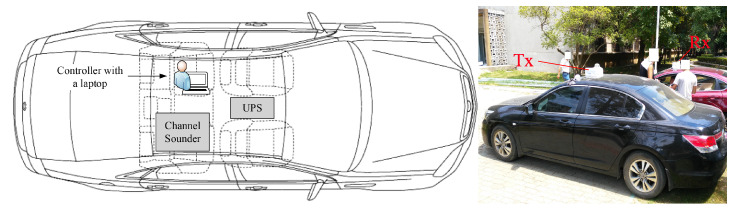
Example of the installation for the measurement campaigns.

To conduct the measurement campaigns, we employed two standard sedans: a Fiat and a Honda. Two omnidirectional antennas were mounted on the roof of the Tx car and Rx car. Cables were passed through gaps created by the sunroof, then connected to the sounders inside test cars. Additionally, the device needed to use GPS synchronization settings before performing the measurement experiment. The installation instructions are shown in Figure 3. When conducting campaigns, we placed the channel sounder on the back seat of the car, which was powered by an uninterruptible power supply support 12 V DC. During the measurement, we used two laptops to control them and stored the raw complex data—the time-variant channel transfer function (CTF) centered at 5.9 GHz—on the laptops in real-time. The latitude and longitude together with velocities were also stored.

### 2.2. Measurement Description

#### 2.2.1. Car-Following Scenarios

Congestion, low-speed: Traffic jams often occur in large cities in China. In our first measurements, the two vehicles moved slowly in a traffic jam with slow speeds ranging from 0 to 5 m/s. This was a road through a dense urban area. Because the traffic in both directions converges from viaducts to this road, it is often congested.

Non-congested, medium speed: The traffic traveled smoothly on the opposite section of the congested road mentioned above. To compare the channel characteristics under different vehicle densities in the same environment, we conducted a measurement campaign on the opposite section of the same road. The two test cars maintained an average speed of approximately 10.3 m/s.

**Figure 4:**
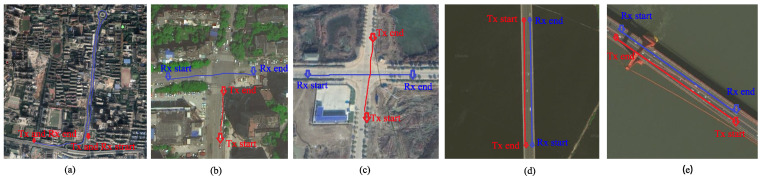
Satellite snapshot of the measured scenarios: (**a**) car-following scenarios in a dense urban area, (**b**) intersection in an urban area, (**c**) intersection in a rural environment, (**d**) suspension bridge, and (**e**) beam bridge.

Viaduct, high speed: Viaduct highways are common in large cities in China. Here, we conducted a measurement campaign on a ring road crossing an urban area. There were soundproof walls on both sides of the viaduct, which formed a rich scattering environment. Our measurement vehicles drove in the same direction, with some cars occasionally between the two test cars. The average speed of the Tx vehicle was 17.4 m/s, whereas the Rx vehicle drove at approximately 14.6 m/s. During this time, the Tx vehicle completed an overtaking of the Rx vehicle. The three scenarios are shown in Figure 4a.

#### 2.2.2. Intersection Scenarios

We recorded two kinds of measurements for the intersection scenarios, i.e., urban and rural areas. In these two scenarios, both test vehicles moved toward the intersection from perpendicular directions.

Urban: For the urban environment, the scenario was located on a noisy block surrounded by many tall buildings, similar to an urban canyon. When the measurement campaign started, the Tx car was blocked at the crossroad because of the traffic light, and the Rx car went straight through the intersection at a speed of approximately 8.33 m/s.

Rural: In contrast to the rich scattering environment in the urban area, there were few buildings located in the rural area where the measurement campaigns were conducted. There were no other vehicles around the two measurement cars. Consequently, the two measurement cars moved at an average speed of 11.1 m/s. The two intersection scenarios are shown in Figure 4b,c.

#### 2.2.3. Opposite Traveling Scenarios

Suspension bridge: In this scenario, the Tx and Rx vehicles traveled on a 3.4 km-long suspension bridge across the Yangtze River (the third-longest river in the world). The two measurement vehicles drove in opposite directions with an average relative speed of 36.92 m/s. Then, the two measurement vehicles met on the middle of the bridge during our measurement campaign. The bridge is suspended by many steel cables that are connected to three high bridge towers.

Beam bridge: For this measurement, we chose a beam bridge on an urban lake. Unlike the structure of the suspension bridge, the bridge is a rigid continuous beam bridge with a length of 1.3 km. Two cars drove in the opposite directions at different speeds: the Tx vehicle had an average speed of 18.5 m/s; the Rx vehicle moved at an average speed of 21.7 m/s. There were only short guardrails on the bridge side, and there were no towering steel cables. The two opposite traveling scenarios are shown in Figure 4d,e.

## 3. Local Region of Stationarity Calculation

Due to the dynamic changes in the scatterers in the vehicle-to-vehicle wireless channel and the high-speed motion of the transmitting and receiving stations, the wireless channel parameters exhibit time-varying non-stationary characteristics. To accurately quantify the variation characteristics of the wireless channel parameters, it is necessary to present a window for the analysis of the V2V wireless channel parameters, that is, the local statistical interval. Only the channel parameters extracted in the statistical interval have statistically significant physical meanings.

**Figure 5:**
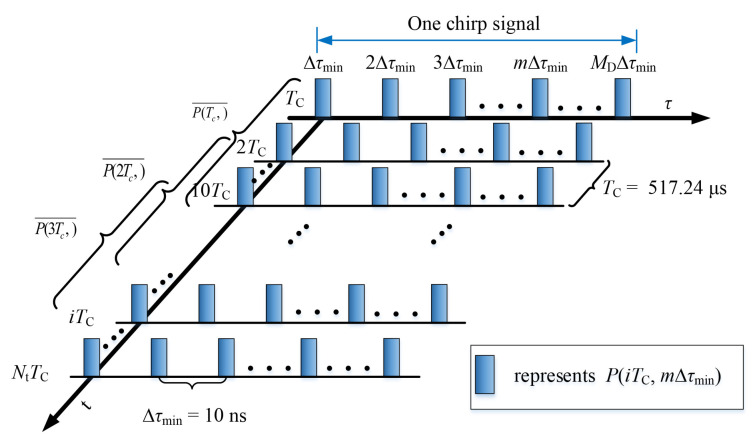
Algorithm schematic diagram.

To determine the LRS, we needed to analyze the raw data derived from the measurements. The raw data were stored on a laptop in real-time in the form of discrete time-varying transmission functions $H\left(iT_{C},n\Delta f\right)$ in the frequency domain when conducting the measurement campaigns. After the inverse discrete Fourier transform (IDFT) was applied, we derived the discrete channel impulse response (CIR) $h\left(iT_{C},m\Delta \tau_{\min}\right)$ (shown in Equation (1) and Figure 5). The bandwidth of the transmitted signal was B = 100 MHz, so the channel delay resolution $\Delta \tau_{\min}=1/B=10$ ns.

(1) $$h\left(iT_{C},m\Delta \tau_{\min}\right)=F^{-1}\left[H\left(iT_{C},n\Delta f\right)\right]$$

where $H\left(iT_{C},n\Delta f\right)$ represents the discrete time-varying transmission function, $i\in \left\{1,2,3,\ldots ,N_{t}\right\}$ is the time index, $N_{t}$ describes the total chirp number of the measured data, Δf expresses the frequency sampling resolution, $n\in \left\{1,2,3,\cdots ,I_{f}\right\}$ denotes the frequency index, and $I_{f}$ is the number of sampling points in each chirp signal in the frequency domain. $m\in \left\{1,2,3,\cdots ,M_{D}\right\}$ represents the time-delay index and $M_{D}=2560$ denotes the number of sampling points in each chirp signal in the time-delay domain. Because a channel sounder was employed in many other measurements in various scenarios (see, for example, [41,42]), the CIR calculation can be found in [42].

The instantaneous PDP is used to acquire the LRS. For the discrete CIR, it is expressed as $h\left(iT_{C},\;m\Delta \tau_{\min}\right)$, and the discrete instantaneous PDP (IPDP) can be derived as shown in Equation (2).

(2) $$P\left(iT_{c},m\Delta \tau_{\min}\right)={\left|h\left(iT_{C},m\Delta \tau_{\min}\right)\right|}^{2}$$

To remove the impact of fast fading, we average the particular amount of instantaneous PDP, as shown in Equation (3).

(3) $$\bar{P\left(iT_{C},m\Delta \tau_{\min}\right.})=\frac{1}{N}\sum_{i}^{i+N-1}{\left|h\left(iT_{C},m\Delta \tau_{\min}\right)\right|}^{2}$$

*N* is the width of the sliding window to calculate the averaged PDPs shown in Figure 4. The averaged PDPs are used in Equation (4) to compute the temporal PDP correlation coefficient $c\left(iT_{c},\Delta t\right)$.

(4) $$c\left(iT_{C},\Delta t\right)=\frac{\int P\left(iT_{C},m\Delta \tau_{\min}\right)\cdot P\left(iT_{C}+\Delta t,m\Delta \tau_{\min}\right)d\tau }{\max\left\{\int P{\left(iT_{C},m\Delta \tau_{\min}\right)}^{2}d\tau ,\int P{\left(iT_{C}+\Delta t,m\Delta \tau_{\min}\right)}^{2}d\tau \right\}}$$

Here, the LRS presents the geographical area beginning with the maximum value of Δx in Equation (5).

(5) $$d_{LRS}\left(iT_{c},\Delta t\right)=max\left\{{\Delta x|}_{c\left(iT_{c},\Delta t\right)\geq c_{th}}\right\}$$

where Δx=v·Δt. In particular, we define the threshold $c_{th}$ = 0.2 [43].

## 4. Measurement Evaluation and Data Analysis

### 4.1. Car-Following Scenarios

**Figure 6:**
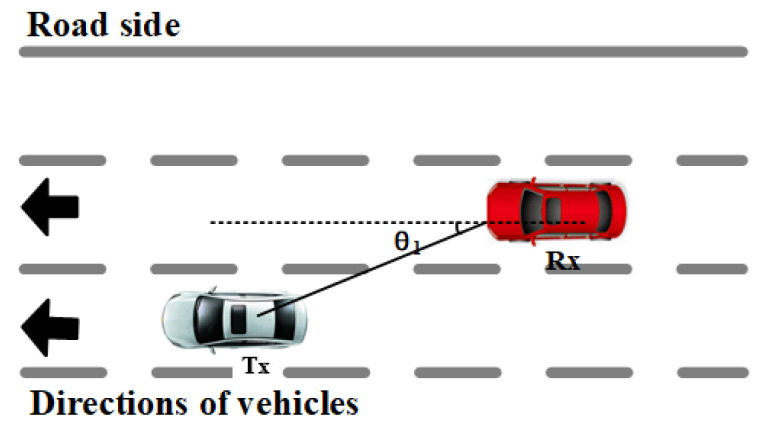
Schematic diagram of the relative speed of two cars traveling in the same direction.

The two test vehicles drove in adjacent lanes, but their speed varied with the traffic status (Figure 6). To analyze the relationship between the relative speed and the stationary time, the calculation formula of the relative speed of the two vehicles is given according to Figure 6. Equation (6) is the calculation expression of the relative speed, where $v_{tx}$ is the speed of the Tx car, $v_{rx}$ is the traveling speed of the Rx car, and $\theta_{1}$ is the angle between the LoS path of the two cars and the traveling direction of the Rx car. From Equation (6), the relative speed is the least when the two cars are parallel.

(6) $$\Delta v=\left(v_{tx}-v_{rx}\right)\cdot \cos\theta_{1}$$

#### 4.1.1. Congestion, Low-Speed Scenario

**Figure 7:**
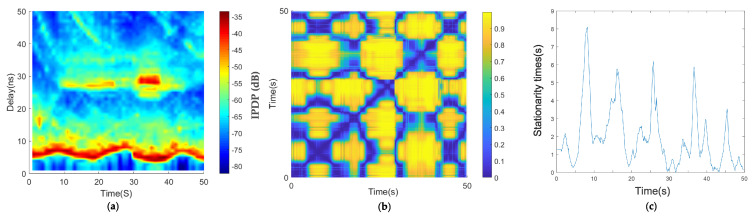
Results of the car-following, congestion, low-speed scenario: (**a**) average PDPs, (**b**) temporal PDP correlation coefficient, and (**c**) stationarity time estimate.

By analyzing the measured data, we derived the channel characteristics under a low-speed scenario in the car-following environment (Figure 7). In Figure 7a, the horizontal axis is time, which represents the measured time, and the vertical axis is the delay. Each slice perpendicular to the horizontal axis represents the power delay spectrum of the absolute delay of the channel at each time. The PDP diagram in the time domain was obtained by combining multiple time-delay spectra. Figure 7a shows the change in the distance between two vehicles, the channel multipath characteristics of the environment of two vehicles at different times, and the evolution characteristics of the channel multipath. Figure 7a shows that the main paths of the two vehicle’s channels were basically parallel to the x-axis with some small fluctuations, which indicates that the absolute delay of the two vehicles experienced slight changes. It was also inferred that the two vehicles were closer and the relative distance changed little, which is consistent with the low-speed traffic jam scene. Throughout the whole process, the PDP diagram shows that the reflection path was relatively rich, and there was an obvious reflection path at the absolute time delay of about 30 ns from 10 to 40 s.

Figure 7a,b shows the average PDPs and the coherence region of the car-following congested low-speed scenario. As shown in Figure 7c, there are multiple large areas of stationary times in the diagonal direction. The algorithm threshold selected in this paper is 0.2, which has been mentioned in Section 3.

There are several large measured stationary times in the car-following, congestion, low-speed scenario in Figure 7c; the maximum value was 8 s, and the larger values were in the range of 4–6 s. Because this is a traffic jam scenario, when the two cars stopped at the same time, the two vehicles were in a relatively static state; therefore, the stationary time increased, and when the relative speed of the two vehicles was large, the measured stationary times decreased. The calculation result is consistent with the actual situation.

#### 4.1.2. Medium-Speed Scenario

**Figure 8:**
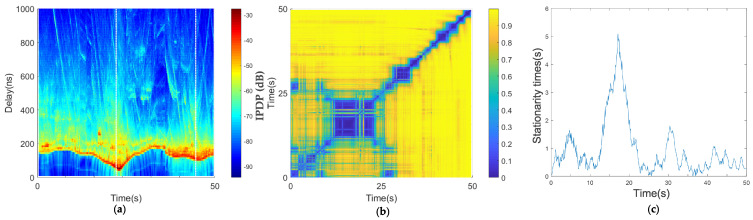
Results of car-following, medium-speed scenario: (**a**) average PDPs, (**b**) temporal PDP correlation coefficient, and (**c**) stationarity time estimate.

Figure 8a shows that the main path of the PDP was parallel to the x-axis most of the time, and the absolute delay was about 180 to 150 ns, forming a V shape at 17 s. The V shape of the main path in the PDP indicates that the absolute delay of the two vehicles first decreased and then increased, which may be a meeting or overtaking. According to the GPS information, at this moment, the two vehicles completed an overtaking, and the two vehicles experienced the process of approaching, driving in line, and then moving away. Therefore, we can see that the stationary time in Figure 8c gradually increases and then decreases. Additionally, throughout the whole process, we can see from the PDP that the scattering path is relatively rich in this environment.

Figure 8b shows a large blue area in the coherence region of the medium-speed scenario. During this period, the two cars were overtaking. During this time, the speed difference was not large. As shown in Figure 8c, the window of stationary times during this period was 5 s. However, in Figure 8c, the window of stationary times is smaller than that of the traffic jam scenario. Since the environment of the two scenarios was almost the same, we inferred that the important factors affecting the window of stationary times are the absolute speed and relative speed, and the relative speed is shown in Figure 6 and Equation (6). When the distance between the two cars is relatively large, the angle between the two vehicles is far from 90 degrees, and the relative speed is large. When the two cars are close, the angle is large and the relative speed is small.

#### 4.1.3. The High-Speed Scenario on the Viaduct

**Figure 9:**
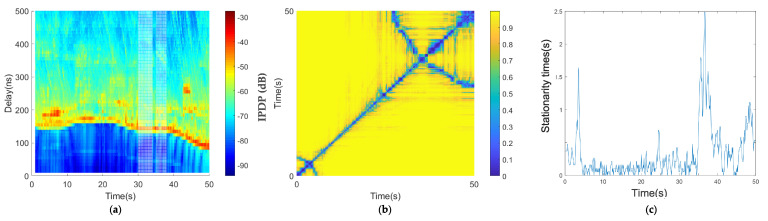
Results of the car-following, high-speed scenario on the viaduct: (**a**) average PDPs, (**b**) temporal PDP correlation coefficient, and (**c**) stationarity time estimate.

From Figure 9a, in the PDP of the high-speed car-following scene on the viaduct, the absolute delay of the two cars ranged from 100 to 150 ns, which indicated that the distance between the two cars was about 30 to 45 m. The absolute time delay of the two vehicles did not change much during 0 to 40 s, indicating that the relative distance between the two vehicles was constant. At this time, the two vehicles traveled in the same direction and the relative speed was constant. However, the absolute time delay of the two vehicles gradually decreased after 40 s, indicating that the distance between the two vehicles was decreasing. It can be seen from the PDP that the reflection path was rich in this environment, which was caused by the buildings on both sides of the viaduct and the soundproof wall.

As shown in Figure 9b, the coherence region of the high-speed scenario is small. Figure 9c shows that the stationary time of 1.5 s appeared in 3 s and that the stationary time of 2.5 s appeared in 37 s compared with the previous two car-following scenarios. The stationary times further reduced due to the increase in the relative speed of the two vehicles. The two regions with larger stationary times, 4 and 37 s, can be seen from the average PDPs in Figure 9a, where the speed of the two cars are almost the same.

### 4.2. Intersection Scenarios

**Figure 10:**
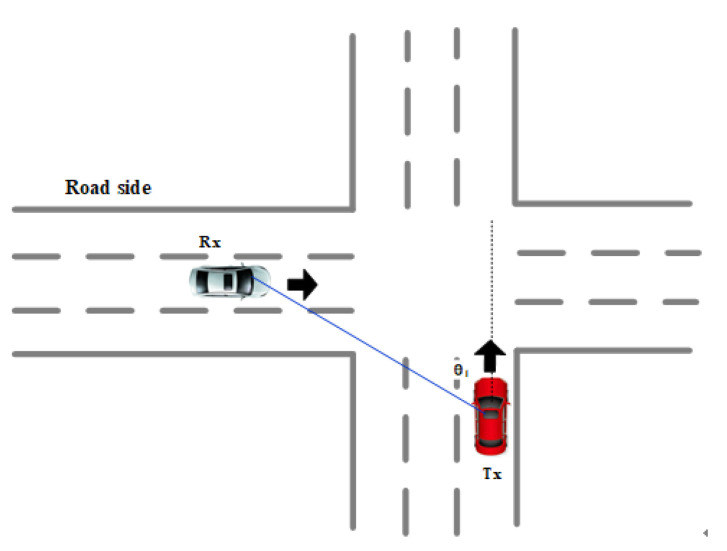
Schematic diagram of the relative speed of the two cars in the orthogonal direction.

The two testing cars were moving like that shown in Figure 10. The relative speed under the intersection scenario is shown in Equation (7), where $v_{tx}$ is the speed of the Tx vehicle, $v_{rx}$ is the traveling speed of the Rx vehicle, and $\theta_{1}$ is the angle between the LoS path of the two vehicles and the traveling direction of the Tx vehicle.

(7) $$\Delta v=v_{rx}\cdot \sin\theta_{1}+v_{tx}\cdot \cos\theta_{1}$$

#### 4.2.1. Urban Intersection Scenario

**Figure 11:**
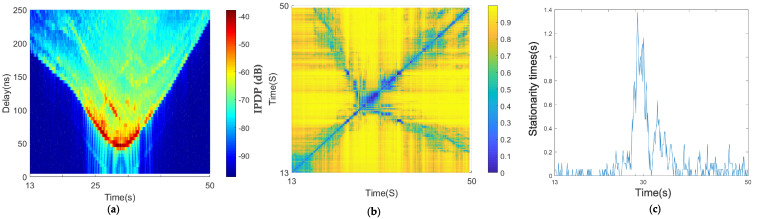
Results of the urban intersection scenario: (**a**) average PDPs, (**b**) temporal PDP correlation coefficient, and (**c**) stationarity time estimate.

The average PDPs of the intersection scenario are V-shaped in Figure 11a: one vehicle remained stationary and the other car moved in a direction perpendicular to the direction of the stationary vehicle. As the moving vehicle traveled, its speed relative to the stationary vehicle increased from large to small.

The time when the two vehicles were closest was 30 s; the distance between the two vehicles was about 15 m at this moment. Figure 11a shows that there were abundant scattering paths in the urban area, and there was a strong reflection path in the range of 25–30 s. The absolute delay of this reflection path relative to the main path was about 40 ns. The correlation matrix shows that the diagonal part is in the shape of a blue line, and there is an obvious box at about 30 s (Figure 11b); due to the minimum distance between the moving vehicle and the stationary vehicle, the angle reached 90 degrees and the relative speed reaches the minimum. Figure 11c shows that the maximum steady time at this moment was 1.4 s, whereas the steady time was short during the other moments because the two vehicles were continuously approaching or traveling away from each other; the stationary time was less than 0.1 s, and the maximum was only 0.2 s.

#### 4.2.2. Suburban Intersection Scenario

**Figure 12:**
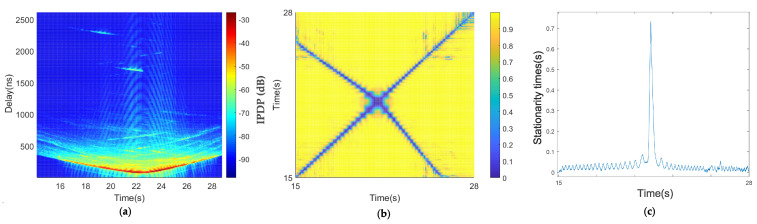
Results of the suburban intersection scenario: (**a**) average PDPs, (**b**) temporal PDP correlation coefficient, and (**c**) stationarity time estimate.

As shown in Figure 12a, the average PDPs at the crossroads of the suburbs present a more pronounced V shape because both vehicles were moving in the suburban scenario, and the relative speed was larger than that in the urban area. Figure 12a shows that there were many scatterers in this environment. There were still reflection paths at the absolute time delays of 1800 and 2300 ns, but the existence time of the reflection path was not long. Because there were few vehicles in the suburbs, the speed of vehicles in this scene was faster than that in the urban intersection scene. Figure 12b shows that faster speed led to smaller diagonal width and a larger blue box appeared at about 20 s. According to the analysis in the previous paper, the box is the time when the distance between two vehicles is the shortest. At this time, the maximum stationary time was 0.7 s, and the stationary time excluding the maximum stationary time was approximately 0.02 s, as shown in Figure 12c.

The relative speed relationship is shown in Figure 10. When the two cars met, the angle suddenly became 90 degrees, which is consistent with the result for the stationary time.

### 4.3. Opposite Traveling Scenario

**Figure 13:**
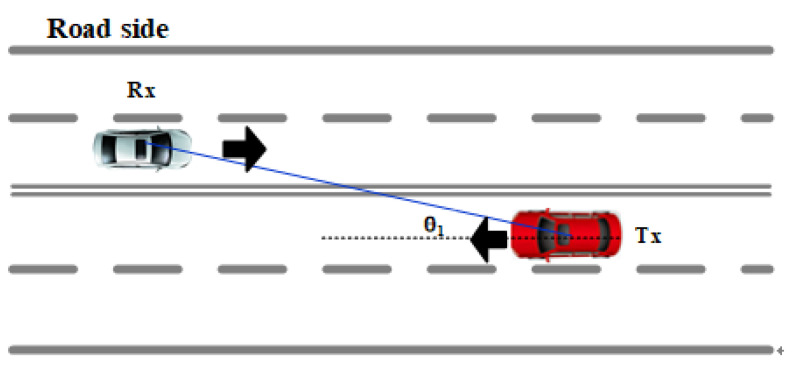
Schematic diagram of two cars traveling in opposite directions.

Figure 13 depicts a schematic diagram of the two test cars moving in opposite directions. When the two vehicles were closest, the instantaneous relative speed of the two vehicles was 0. A detailed description of the measurement campaign is provided in Section 2.

(8) $$\Delta v=\left(v_{tx}+v_{rx}\right)\cdot \cos\theta_{1}$$

#### 4.3.1. Beam Bridge Scenario

**Figure 14:**
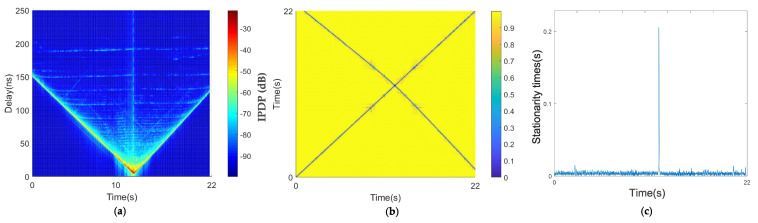
Results of the opposite traveling beam bridge scenario: (**a**) average PDPs, (**b**) temporal PDP correlation coefficient, and (**c**) stationarity time estimate.

Figure 14 depicts the result of two cars driving in opposite directions on a beam bridge. The bridge was about 10 m above the water, and the water was relatively open. The environment around the bridge was very open, and there were almost no objects within a certain distance. The only obstacle was the guardrail of the bridge, with a height of 1.3 m, which is lower than the height of the test vehicle. Thus, the scattering path was less than that in the other scenes, as shown in Figure 14a. The shape of the PDPs presents a typical V shape, which means the two vehicles were in different lanes and driving in opposite directions, completing a rendezvous process. As shown in Figure 14b, the diagonal of the correlation matrix is almost a straight line, which indicates that the two vehicles were rapidly approaching or moving away from each other at the non-meeting time, and the steady window is very small. At the moment when the two cars met, it was about 14 s, and the corresponding steady-state time presented like a pulse, which indicated that the two cars quickly drove apart after the instant meeting. The low relative speed at the moment of meeting resulted in the steady-state time suddenly increasing, and the stationary time at the moment of meeting was 0.2 s.

#### 4.3.2. Suspension Bridge Scenario

**Figure 15:**
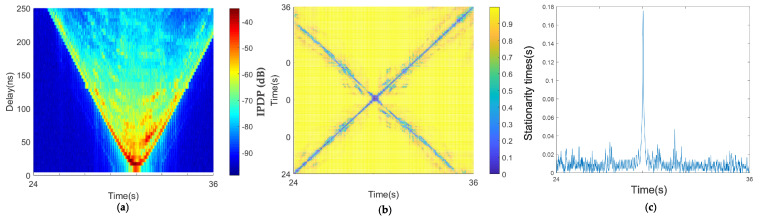
Results of the opposite traveling suspension bridge scenario: (**a**) average PDPs, (**b**) temporal PDP correlation coefficient, and (**c**) stationarity time estimate.

The PDP of the suspension bridge scene is still V-shaped, as shown in Figure 15a. The scattering path is much greater than that in the beam bridge scene. By comparison, we found that there were no scatterers higher than the vehicle height on the beam bridge, whereas there were a large number of scatterers higher than the vehicle height on the suspension bridge. The metal suspension tower and steel cables are good reflectors of electromagnetic waves and can cause abundant scattering, which produced the difference in the scattering environment between the beam bridge and suspension bridge scenarios. The diagonal of the correlation matrix of the suspension bridge is almost a straight line, which is similar to that of the beam bridge scene (Figure 15b). However, the diagonal in Figure 15b is rougher than that in Figure 14b, which was produced by the difference in the steady-state matrix caused by the scattering of the steel cables.

The coherence region of the opposite-traveling-direction suspension bridge scenario is also almost a straight line in Figure 15b, similar to the beam bridge scenario. The measured stationary times in Figure 15c show that the largest stationary time was 0.175 s when the two cars met, which is smaller than in the beam bridge scenario. According to the speed information, this is because the relative speed of the two cars in the scenario was lower. When the two cars did not meet, the stationary times were also small, but they are larger than the beam bridge scenario, and the fluctuation is more severe. This is because the suspension bridge had many steel cables, forming many scatterers. This is the difference between the two bridge scenarios. A schematic diagram of the relative speed of the two cars traveling in opposite directions is shown in Figure 12 and by Equation (8) The minimum relative speed appears when the two cars meet.

### 4.4. Statistical Analysis

**Figure 16:**
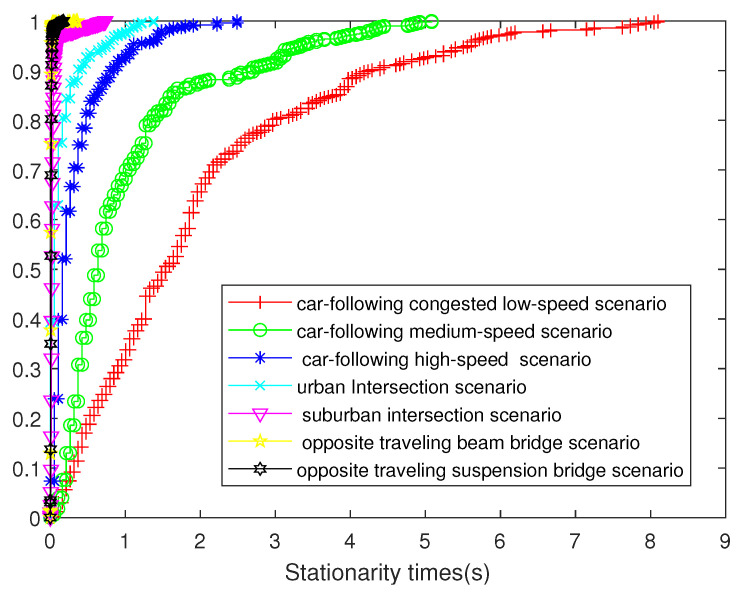
CDF of stationary times of the seven scenarios.

Based on the measured data in the previous sections, the stationarity time was derived by calculating the temporal PDP correlation coefficient. To better demonstrate the statistical characteristics of the results, the statistical results are elaborated in this section. Table 1 shows the maximum, mean, and standard deviation of the measured stationary times in seven scenarios, and Figure 16 shows the cumulative distribution function (CDF) of the stationary times of the seven scenarios. The most relevant factors affecting the measured stationary times’ size are the relative speed and absolute speed. Among them, the measured stationary times’ size was the largest in the same-direction driving scenario because the relative speed was the least. The absolute speed in these three scenarios was larger, and the measured stationary times were shorter. This conclusion is the same in paper [25], in which the measured average value of the window of stationary times in the same direction was 1.4 s, while the window of stationary times in the opposite direction was only 0.023 s, which is larger than the value in this paper because the average speed of 8.3 m/s in Scenario 3 is faster than in our measurement. For instance, traffic jams are common in China; the velocity in the congestion scenario ranged from 0.05 to 3.76 m/s. Notably, the relative speed in our measurements of the car-following congestion scenario was quite slow, because the two test cars were stuck in a traffic jam during the campaign. Thus, the mean value of the stationary time of 1.9419 s is much longer than that in [25]. Additionally, the length of the measurement in [25] was only two seconds, and the relative speed change in the same direction was very small. We used a longer measurement period, so the result is more informative.

Similarly, the average velocity of 13.89 m/s in the urban environment in [39] resulted in a coarse stationarity time of 0.685 s. This speed is similar to what we measured in the car-following high-speed scenario. However, the rich scattering environment caused by the soundproof wall on the viaduct led to a shorter mean stationary time (0.3207 s). Additionally, the complex scattering environments differ from the measurements recorded in European and American cities, which is an interesting point worth studying.

In the scenario with two intersections, in the scene with the intersection in the urban setting, one car was stopped and one car was moving; in the scenario with the suburban intersection, both cars moved. Therefore, suburban intersections exhibit a larger relative speed and have smaller windows of stationary times and average stationary times.

For the opposite driving scenario, the authors of [25] conducted a measurement at a speed of 25 m/s. However, the authors of [39] only considered the same-direction driving situation. Specifically, in the window of stationary times in the opposite direction, we did not find the same spikes in stationary times as found in [25]. This is because the resolution of the window of stationary times in this study was large. In the two opposite-driving-direction scenarios, the maximum or average value of the stationary times window was very small, i.e., the mean value of the beam bridge scenario only reached 0.0041 s. The relative speed of the two cars in the suspension bridge scenario was slightly lower than in the beam bridge scenario, so the maximum value of the stationary times was slightly larger. In the scenario of the beam bridge, there was no stay cable, so when the two cars did not meet, the stationary times fluctuated little, and the suspension caused the stationary times to fluctuate due to the existence of the stay cable, resulting in the suspension bridge scenario exhibiting an average LRS length slightly larger than that of the beam bridge scenario.

## 5. Conclusions

In this study, we conducted a series of continuous V2V extensive measurement campaigns to obtain a large amount of data. Because of the particularity of V2V communication, the two communication terminals always move rapidly in various environments. Due to the unique motion mode of a group of vehicles, the V2V channel is characterized as quickly time-varying. The channel of V2V is not a traditional stationary channel, but a non-stationary channel. Therefore, the main significance of this study is analyzing the non-stationary characteristics of the V2V channel and laying the foundation for research to improve V2V communication systems. To study the stationary time of different vehicle motion states, we used seven different sets of V2V measurement data, including three different traffic flow conditions in the same direction. In addition, urban and suburban conditions in the case of perpendicular driving and two conditions of opposite-direction driving on different bridges were evaluated. Then, we extracted the PDPs from the measured data. We obtained considerable amounts of information from the PDP diagram. In the scenario of two vehicles driving in the same direction in an urban area, the reflection path with strong power was often found with a short delay (100 ns), and there were rich reflection paths with longer delays (500–1000 ns). The reflection paths showed obvious appearance–disappearance phenomena; regardless of distance, the reflection paths appeared randomly and then disappeared after a while. In the scenario with two vehicles driving perpendicular to each other, there were also strong reflection paths in the near distance in the urban area, and there were significantly more reflection paths in the urban area than in the suburbs. As shown in Figure 12a, two reflection paths with appearance–disappearance characteristics were observed at a delay of 1700–2200 ns, which indicated that in relatively open scenarios, the buildings at a long distance formed reflection paths with appearance and disappearance characteristics. The PDP comparison between the beam bridge and the suspension bridge showed that the reflection path of the suspension bridge was much richer than that of the beam bridge; the scattering environment of the suspension bridge was similar to that of the urban area. The metal suspension cable plays the role of reflecting electromagnetic waves like the buildings in the urban area, which form a large number of scattering paths in the continuous delay domain with certain appearance and disappearance characteristics.

Based on the average PDPs, the LRS method was explored to analyze non-stationary V2V radio channels. The results showed that the factors affecting the stationary times are, in turn, the direction of the two vehicles, the relative speed of the two vehicles, and the environment. In the seven scenarios, the maximum stationary times ranged from 0.175 to 8.0995 s, and the average stationary times ranged from 0.0103 to 1.9419 s. With a suitable stationary time, we obtained a series of channel characteristics parameters, including the small-scale fading distribution, average Doppler, RMS Doppler, average delay, and RMS delay. Changes in these channel characteristic parameters affect the performance of the communication system, including the information transmission rate and the information error rate.

## 6. Discussion

Electric vehicles and automobile intelligence have been the direction of automobile development in recent years. With the increase in people’s environmental protection requirements, tram replacement is a future trend. With the success of smartphones, another development trend in electric vehicles is intelligence and information. At present, vehicle communication uses the central communication method, and V2V communication is developing toward non-central communication. Different from traditional cellular communication, the power of vehicle communication equipment is low, the topology of the vehicle is changeable, and the terminal may be accessed repeatedly. In V2V communication, the transceiver is moving rapidly, and the channel has typical non-stationarity. The antenna height of the car-to-car communication transceiver is low and is considerably affected by environmental factors. However, the reliability requirement of V2V communication is high, and its delay must be low.

The main contributions of this study include obtaining a large amount of high-precision channel propagation data from various road conditions and the analysis of the data. Compared with similar studies, the data in this paper are more abundant, which more accurately describe the characteristics of stationary time under a variety of driving conditions, including the stationary time at different times in the same scenario. In particular, the test data in this paper are long, up to 50 s. In such a long period, the test vehicles repeatedly overtook one another, and the two test vehicles mixed with other vehicles many times, so the single-scenario test statistical data in this paper cover more cases. The movement conditions in vehicle-to-vehicle communication change rapidly. Two vehicles may be driving in the same direction in a previous moment, and they may be driving in the opposite direction in the next moment, which leads to large changes in the stationary time and has a strong impact on communication. Future research methods should involve recording multiple measurements and performing comparisons under different speed conditions in the same environment.

Overall, the measurement and analysis results showed that the relative velocity of the vehicle is the most important factor. In principle, we should also deeply explore the influence of different environments on the stationary time in the same scenario and at the same speed. However, the single variable principle will be hard to achieve because of the difficulty of the measurement test organization and the public road scenarios. The environment has little effect on the stationary time in the case of moving at the same speed.

Taken together, these results indicate the importance of time-scale selection for analyzing statistical channel characteristics. Although important discoveries were revealed by these studies, there also exist some limitations. Firstly, the channel propagation data can be further enriched, including more combinations of environments and vehicle driving states. Secondly, the measurement and data analysis of higher frequency bands and a MIMO system will be included in our next study.

## Figures and Tables

**Table 1:** Stationary times for the seven scenarios.

Scenarios	Stationary Time		
	Max (s)	Mean (s)	Standard Deviation
Car-following, low-speed scenario	8.0995	1.9419	1.6587
Car-following, medium-speed scenario	5.0821	0.987	1.0112
Car-following, high-speed scenario	2.4881	0.3207	0.3932
Urban intersection scenario	1.3764	0.1348	0.2386
Suburban intersection scenario	0.7336	0.0359	0.0746
Beam bridge scenario (opposite)	0.2089	0.0041	0.008
Suspension bridge scenario (opposite)	0.175	0.0103	0.0134

## Data Availability

Data is contained within the article.

## Abbreviations

The following abbreviations are used in this manuscript:

V2V	Vehicle to Vehicle
LRS	Local Region of Stationarity
PDPs	Power Delay Profiles
LoS	Line of Sight
NLoS	Non-Line-of-Sight
LSF	Local Scattering Function
SNR	Signal Noise Ratio
MSE	Mean Square Error
MPCs	Multi-path Component
WSSUS	Wide-Sense Stationary Uncorrelated Scattering
CCF	Channel Correlation Function
CMD	Correlation Matrix Distance
IDFT	Inverse Discrete Fourier Transform
CIR	Channel Impulse Response
CDF	Cumulative Distribution Function
